# Trait-Based Selection of Seeds Ingested and Dispersed by North American Waterfowl

**DOI:** 10.3390/plants14131964

**Published:** 2025-06-26

**Authors:** Bia A. Almeida, Mihai Costea, Giliandro G. Silva, Leonardo Maltchik, Susan E. W. De La Cruz, John Y. Takekawa, Andy J. Green

**Affiliations:** 1 Department of Conservation Biology and Global Change, Estación Biológica de Doñana (EBD), Consejo Superior de Investigaciones Cientifícas (CSIC), 41092 Sevilla, Spain; bialmeida182@gmail.com; 2 Department of Biology, Wilfrid Laurier University, Waterloo, ON N2L 3C5, Canada; mcostea@wlu.ca; 3 Graduate Program in Biology of Continental Aquatic Environments, Federal University of Rio Grande—FURG, Rio Grande 96203-900, Brazil; giliandrog@gmail.com (G.G.S.); maltchikleo@gmail.com (L.M.); 4 U.S. Geological Survey, Western Ecological Research Center, San Francisco Bay Estuary Field Station, Moffett Field, CA 94035-0158, USA; sdelacruz@usgs.gov; 5 Suisun Resource Conservation District, 2544 Grizzly Island Road, Suisun City, CA 94585-9539, USA; jtakekawa@suisunrcd.org

**Keywords:** seed dispersal, foraging guild, functional trait, endozoochory, long-distance dispersal, Anatidae, migratory birds, feeding behavior

## Abstract

There are few studies on the extent to which waterfowl select plant food compared with what is available in wetland ecosystems. We used a new dataset on the presence of seeds in the alimentary canal or feces to identify flowering plant species whose seeds are ingested by North American ducks or geese. These data are a proxy for dispersal interactions because an important fraction of ingested seeds survives gut passage and is dispersed by endozoochory. We compared the plant traits of species whose seeds were ingested with those of species on the U.S. Department of Agriculture National Wetland Plants List (NWPL). Using a global dataset on plant form and function and chi-squared tests, we compared four categorical traits (moisture requirements, growth form, plant height, and seed mass) between species whose seeds are ingested by North American ducks and geese with the NWPL. Our analyses identified significant differences between the trait distributions of plants whose seeds were ingested by waterfowl guilds and those of the NWPL. Geese and ducks (except whistling ducks) ingested more aquatic and semiaquatic plant species than expected from the NWPL. All guilds except sea ducks ingested more herbaceous graminoids and fewer shrubs or trees than expected. Diving ducks interacted with fewer of the taller plants (>5 m) than expected, but otherwise plant height distributions did not differ from those expected. All waterfowl guilds ingested more species of intermediate seed size (1–10 mg) and fewer species of the smallest (<0.1 mg) or largest (>100 mg) size categories than expected. These results help to explain the role of the long-distance dispersal of seeds by migratory waterfowl in plant biogeography and how plant distributions are likely to respond to global change.

## 1. Introduction

Plant seeds form a major component of the diet of many North American waterfowl (Anatidae), and wetlands used for hunting are often managed to boost seed production [1,2]. Most of these bird species undergo long-distance migration, and an important fraction of these seeds survives gut passage, being dispersed through “endozoochory” between the point of ingestion and of egestion in feces [3,4,5,6]. Hence, migratory waterfowl are important vectors for the long-distance dispersal (LDD) of wetland flowering plants [7,8]. Through seed dispersal, waterfowl in North America provide a major ecosystem service, ensuring connectivity and gene flow between plant [9].

The large migratory populations of many waterfowl species [10] provide enormous potential for LDD, and an individual bird can often disperse hundreds of seeds per day [11,12]. Even during non-migratory periods, the maximum seed dispersal distances provided by waterfowl endozoochory exceed those of most frugivorous terrestrial birds and greatly exceed those provided by abiotic mechanisms such as wind (anemochory) or water (hydrochory) dispersal, exchanging seeds between habitats that are otherwise unconnected [7,13,14]. Hence, waterfowl allow plants to respond to climate change or land-use change [15,16]. Dispersal on the outside of waterbirds (epizoochory) is also important for some plants [17], but endozoochory is far more frequent [8,18].

However, our knowledge of *which* of the plants occurring in and around wetland habitats are ingested and dispersed by waterfowl remains limited. Classical dispersal syndromes based on seed morphology [19,20] do not recognize the endozoochory of any plants that lack fleshy fruit, and thus ignore the reality that waterfowl are frequent dispersers of plant species from across a range of dispersal syndromes [21]. Recently, Almeida et al. [22] reviewed the literature to identify plants whose seeds have been recorded in the gastrointestinal tract or feces of North American waterfowl species. Almeida et al. [23] related the traits of different waterfowl species to the traits of the plant species whose seeds they ingest (as a proxy for which seeds they disperse), and found important differences among well-known foraging groups (guilds) such as dabbling ducks, diving ducks, and geese.

In this new study, we go further by considering what plant traits underlie the selection of seeds by waterfowl from those in the environment, i.e., which plant seeds are ingested and which are avoided (by “selection”, we refer to any process of non-random association, which is not necessarily “intentional”). For this, we explored if, from a list of wetland plants in North America, there are traits that differ between plant species whose seeds are recorded in each waterfowl guild and those that are not. Our initial hypotheses were that (I) seeds of aquatic plants are more likely to be ingested and dispersed than terrestrial plants because waterfowl largely feed in an aquatic environment [24,25]; (II) plant height does not limit seed dispersal because birds do not necessarily take seeds from the mother plant and that waterfowl endozoochory is often a secondary dispersal event when seeds are ingested when floating freely or from seed banks after being moved from the mother plant (of any height) to water by wind, rain, or gravity [26]; (III) seeds of herbaceous graminoid (small plant species with a grass-like growth form) species are more likely to be dispersed than those of other growth forms because their seeds are often ingested together with green plant material during herbivorous foraging (i.e., “foliage is the fruit”, [21,27]; and (IV) seeds ingested are those of an intermediate size or mass, as has been reported for European dabbling ducks [28].

We tested these hypotheses for different guilds because they have differences in feeding ecology that could be expected to influence the traits of the plants whose seeds they ingest [29]. Dabbling ducks mainly feed at or close to the surface in and around the shoreline. Diving ducks mainly feed by diving to depths from 40 cm to several meters. Sea ducks are largely marine carnivores that also dive, but often breed in freshwater and may disperse seeds attached to or inside their prey [30]. Geese are herbivores that graze on land or grub around in sediments. Whistling ducks are limited to the southernmost parts of North America, and are relatively understudied.

## 2. Materials and Methods

### 2.1. Literature Search

This study was developed using data gathered by Almeida et al. [22] through a systematic search on all available data regarding the presence of angiosperm diaspores (hereafter “seeds”) within the digestive tract or faeces of North American waterfowl (Anatidae: ducks, geese, and swans). Following Almeida et al. [23] we used this information as a proxy for seed dispersal interactions. We inferred that even the presence of seeds in the esophagus or gizzard is a valid proxy for seed dispersal because a fraction of seeds invariably survives gut passage [4,8,31]. Seed ingestion is the most widespread measure of seed dispersal “quantity” for birds [32], but see [33].

Publications produced until June 2022 were searched using the individual keywords “Plant”, “Diet”, “Food”, “Feeding”, and “Faecal”, together with the scientific and/or common name of each of the 48 waterfowl species that occur in North America according to the IOC World Bird List [34]. We selected studies with samples taken in Mexico, the United States, or Canada that independently specified seed occurrence for each bird species. We only considered plant taxa identified to the species level and removed domesticated plant species that have often been used to attract waterfowl. The complete dataset contained information of 119 studies on 38 bird species. Almeida et al. [22,23] provides more details on how the search was conducted.

We classified waterfowl species into foraging groups (i.e., guilds)—dabbling ducks, diving ducks, geese, swans, sea ducks, and whistling ducks—based on previous studies on waterfowl diet and behavior [24,25]. For our analyses, we used qualitative information on the occurrence of each seed species in the diet of each waterfowl species. We only used diet data from adult or fledged waterfowl, and only recorded the presence or absence of seeds, excluding data on other plant parts. We excluded swans from our analyses due to insufficient data (*n* = 3 studies; [23]). We could not use the abundance or frequency of seeds as an index of endozoochory potential because these data were not reported in a manner comparable between studies (e.g., seeds were rarely counted).

The complete database, including taxa excluded from analyses, can be found in Almeida et al. [22]. Studies on different waterfowl species are often conducted in distinct geographical areas, so the taxonomic composition of gut contents is influenced by the restricted distributions of many plant species. Therefore, we focused on the traits of plants recorded and did not consider the taxonomic differences between those plants ingested by waterfowl species and other plants in the North American flora. In other words, we compared the numbers of plant species with a given trait, but not their identity.

### 2.2. Wetland Plant List and Plant Traits

To investigate how traits explain the likelihood that a given plant in the flora is likely to be dispersed by waterfowl, we needed trait information that could be found not only for our list of ingested plants but also for the plants not found in the waterfowl diet. We used the National Wetland Plant List (NWPL; [35]) for plant taxa in the United States (see the description by Reed [36]). Plant species on this list are not restricted to strictly aquatic plants, but instead are those “that have demonstrated an ability (presumably because of morphological and/or physiological adaptations and/or reproductive strategies) to achieve maturity and reproduce in an environment where all or portions of the soil within the root zone become, periodically or continuously, saturated or inundated during the growing season” [36].

For this comparison, we used the following four traits retrieved from the global spectrum of the plant form and function dataset [37]: (1) plant height (categorical: <0.5 m, 0.5–1 m, 1–5 m, and >5 m); (2) seed mass (categorical in milligrams: <0.1, 0.1–1, 1–10, 10–100, and >100); (3) moisture requirement, based on the type of habitat in which the species naturally grows (categorical: aquatic, semiaquatic, or terrestrial); and (4) growth form, as determined by woodiness and the extent of growth (categorical: herbaceous graminoid, herbaceous non-graminoid, shrub/tree, and other). Using this information, we obtained trait values for ~42% of the taxa on the NWPL plant list and for ~75% of the plant species found in the waterfowl diet. The trait values for the plant taxa found in the waterfowl diet and in the NWPL can be found in the Supplementary Materials. We obtained trait values for 3806 plant taxa on the NWPL wetland list (File S2) and 432 of those identified in the waterfowl diet (File S1). We made no attempt to estimate the missing values (i.e., trait values for species not included in [37]). As trait data were unavailable for 58% of NWPL species, there was a potential bias toward taxa with better-studied traits. Furthermore, we could not rule out the possibility that the smallest seeds are sometimes overlooked in waterfowl diet studies, with further potential for bias.

### 2.3. Data Analysis

To establish how plant traits explain which plants are ingested by waterfowl and which are not, we compared the frequency of occurrence of plant trait categories (considering the four traits retrieved from [37]) ingested by each waterfowl foraging group with the frequency of occurrence of these categories in the NWPL list of North American wetland flora [35]. We used chi-squared tests applied to the counts of plant species belonging to each trait category in the plant list and in the diet of each foraging group. When chi-squared tests were considered to be significant (at α = 0.05), we performed post hoc analyses of the standardized residuals to identify which waterfowl foraging groups differed from the wetland plant list in relation to a certain trait category. We used the chisq.test function from the package stats [38] and the chisq.posthoc.test function from the package chisq.posthoc.test [39] in the R environment, version 4.2.2 [38] to calculate the chi-squared and post hoc tests, respectively. Post hoc analyses used Bonferroni-adjusted standardized residuals (α = 0.05).

## 3. Results

For all four plant traits, we found statistically significant differences in the chi-squared tests comparing the trait distributions of plant species on the NWPL list with those whose seeds were recorded as ingested by different waterfowl guilds (Table 1).

**Figure 1:**
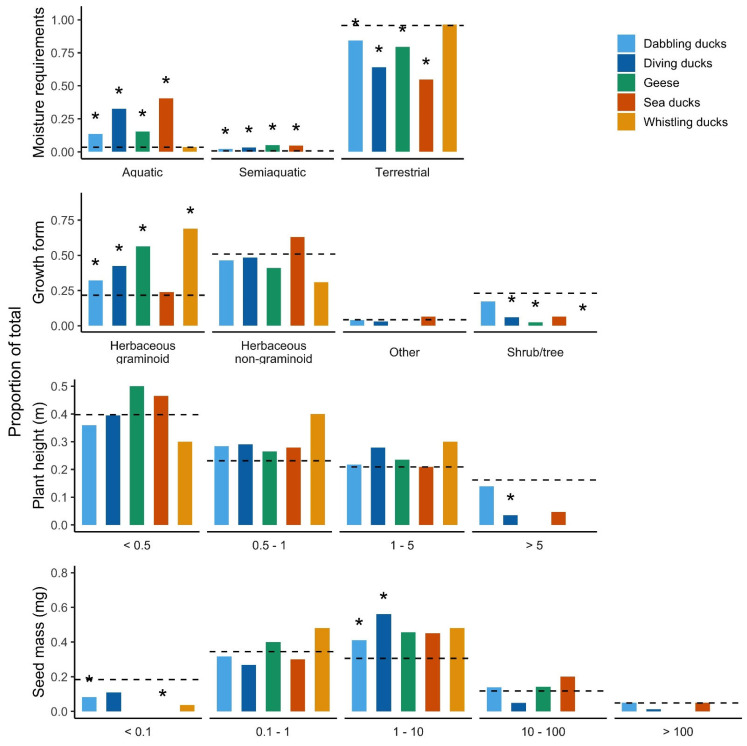
Frequency of occurrence of trait categories for plant species ingested by each waterfowl foraging group (colored columns). These are compared with the frequency of occurrence of these same categories in the NWPL list of North American wetland flora (dashed line) and chi-squared tests. * Significant differences according to post hoc tests. Results for overall chi-squared tests can be found in Table 1. Further details and post hoc tests are given in File S3.

Except for whistling ducks, all waterfowl foraging groups ingested seeds from significantly more aquatic and semiaquatic plant species and fewer from terrestrial plant species than expected based on the number of plant species in North American wetlands belonging to these categories (Figure 1; Tables S1 and S2). This indicates that plant species inhabiting more aquatic environments are more likely to be ingested and transported by most waterfowl species than other wetland plants with lower moisture requirements. The proportion of fully aquatic plants recorded was particularly high for sea ducks and diving ducks. Interestingly, terrestrial plants were dominant in terms of the species number for all foraging groups owing to their greater species richness compared to aquatic plants (Figure 1).

Analyses of growth form revealed that, apart from sea ducks, all waterfowl foraging groups ingested significantly more herbaceous graminoid species than expected from the NWPL list (Figure 1; Tables S3 and S4). Furthermore, all waterfowl groups ingested fewer shrub and tree species than expected; the difference was statistically significant for diving ducks, geese, and whistling ducks (Figure 1; Tables S3 and S4).

For plant height categories, all waterfowl groups ingested seeds from more species of intermediate height (0.5–5 m) than expected as well as fewer species from the highest category with a height of >5 m. However, the only statistically significant effect was that diving ducks ingested seeds of fewer plant species from the highest category than expected (Figure 1; Tables S5 and S6). For other guilds, overall deviations between the observed and expected distributions of height categories were not significant (Table S5). Non-significant trends suggest minimal height-based selection across most guilds, likely due to secondary dispersal mechanisms.

All waterfowl foraging groups ingested seeds from fewer species of the smallest seed size category (<0.1 mg) than expected based on North American wetland flora, a result that was statistically significant for dabbling ducks and sea ducks (Figure 1; Tables S7 and S8). Furthermore, both dabbling and diving ducks ingested seeds of significantly more species with an intermediate mass (1–10 mg) than expected. This was the only one of the four plant traits for which the overall deviation between the observed and expected frequencies was statistically significant for all five foraging groups (Table S7), although post hoc tests found no significant differences for a particular seed size category in the case of geese and whistling ducks (Table S8).

## 4. Discussion

Using a database on seeds in the diet of North American waterfowl produced from an extensive literature search [22], we found marked differences in the distributions of key plant traits between flowering plant species on the NWPL list and species whose seeds were recorded in the gastrointestinal tract or in feces. There is a lack of previous studies comparing seeds ingested by waterfowl with those available in their wetland habitats [1] and, to the best of our knowledge, this is the first study of its kind in the Western Hemisphere. Our results provide novel insights into waterfowl–plant interactions and are of interest from a waterfowl management perspective because they illustrate the broad range of plants whose seeds are included in the diet of North American waterfowl while also suggesting selectivity and detecting some key differences among foraging groups (guilds). On the other hand, our findings reveal how the potential for the LDD of seeds by waterfowl endozoochory depends on these key plant traits. This is particularly important because this dispersal mechanism is overlooked by morphological “dispersal syndromes”, which are defined by an inspection of seed traits [21]. Studies comparing plant traits for species whose seeds are dispersed by waterfowl with those that are present in wetland ecosystems are important to clarify which traits determine the likelihood of waterfowl endozoochory for North American angiosperms in general.

### 4.1. Terrestrial Species Dominate, but Aquatic Species Are Ingested More Often than Expected

Although terrestrial species dominate the NWPL (68% of taxa), most waterfowl disproportionately selected aquatic and semiaquatic plants, highlighting their role as specialized dispersers. Compared with terrestrial species in North America, aquatic plants are less speciose [40,41]. Diving ducks and sea ducks showed the strongest selection for aquatic plants, whereas whistling ducks showed no evidence of such a selection (although this latter group had limited data, *n* = 5 studies). This difference between foraging groups is consistent with the earlier study of Almeida et al. [23], who found that diving ducks and sea ducks ingest and disperse relatively more sublittoral plants (i.e., those found at depths beyond the littoral zone) than other foraging groups. Similarly, it is consistent with general differences in the foraging habitats among waterfowl guilds [24,25].

### 4.2. Growth Form Is Important

We found that plant growth form also influences the chances a plant has of being dispersed by waterfowl. All groups, other than sea ducks, ingested seeds from far more herbaceous graminoid plant species than expected based on their frequency, and most groups ingested fewer shrubs or trees than would be expected based on the number of plant species with these growth forms. Given that fleshy fruited plants tend to be trees or shrubs, this result underlines the importance of the ingestion and endozoochory of dry-fruited seeds by waterfowl in contrast to frugivorous birds [4,5,21]. It is also consistent with the “foliage is the fruit” hypothesis for a mutualistic relationship between herbaceous graminoid plants and waterfowl [21], in which seeds are often ingested together with green foliage.

### 4.3. The Tallest Plants Are Avoided by Diving Ducks

The height of a plant species makes relatively little difference to the likelihood that its seeds are ingested and dispersed, and the difference between heights for the species present in wetlands and the species ingested was only significant for diving ducks (which ingested fewer tall plants than expected). Although most plants whose seeds are ingested and dispersed by waterfowl are non-woody and relatively low in height (<1 m), with the potential for birds to directly ingest seeds from the mother plant, we found little evidence of the selection or avoidance of the tallest plants >5 m. This reflects how seeds are often ingested after they have already been dispersed from the mother plant by gravity, wind, or water [26]. However, the avoidance of seeds from the tallest plants by diving ducks further illustrates the importance of the LDD of dry-fruited aquatic plants by waterfowl, given that these plants are typically assigned to classical dispersal syndromes that imply no potential for LDD (notably unassisted or hydrochory syndromes, [21,23]). Earlier studies that rely on these dispersal syndromes to predict plant dispersal mechanisms have suggested that dispersal distances generally increase with plant height [42]. As such studies ignore dispersal by waterfowl, their conclusions may be incorrect [43].

### 4.4. Waterfowl Prefer Medium-Sized Seeds

All waterfowl ingest seeds from more species with medium-sized (1–10 mg) seeds than expected based on their frequency in the flora, although the difference for this particular size category was only statistically significant for dabbling and diving ducks. Our results also indicate that species from the smallest seed size category of <0.1 mg are rarely ingested, although tiny seeds of <0.1 mg are produced by nearly 20% of the plants listed for North American wetlands. Such small seeds may not be nutritionally attractive but may be well adapted for wind dispersal. The filtering apparatus of ducks may not be well adapted for separating such tiny food items from unwanted sediment and detritus [44]. However, we cannot rule out the possibility that tiny seeds are often ingested by waterfowl, yet are easily digested or frequently overlooked during the seed identification process. As most of the studies reviewed by Almeida et al. [22] focused on diet, the smallest items may sometimes be overlooked. We found no evidence for the selection or avoidance of plants with larger seeds >10 mg or >100 mg. The largest seeds may be hard to ingest or break down in the gizzard.

According to our results, intermediate-sized seeds may be selected by all waterfowl independently of whether they mainly filter seeds from water or sediments or ingest them together with green plant material from the mother plant. These results agree with Soons et al. [28] who used similar data from Europe to show that dabbling ducks ingest seeds of more species with intermediate-sized seeds than expected from European flora, tending to avoid both the smallest and largest seeds. Similarly, Almeida et al. [45] found that seeds with intermediate sizes were ingested by a higher number of waterfowl species in Europe. On the other hand, Almeida et al. [23] found that in North America, dabbling ducks tend to ingest larger-seeded species than other ducks and geese.

### 4.5. Limitations of Our Study

Each study identified from our literature search involved a particular study area, sampling and seed identification methods, and other specific conditions, which could all have influenced the data included in our analysis and, hence, the patterns we observed. For any North American waterfowl species, the seeds they ingest are likely to spatially and temporally vary [24,46]. However, we pooled data from all of North America and at any period of the annual cycle for our analyses. Pooling data across regions and seasons could obscure local or phenological variations in seed ingestion patterns, which should be addressed in future studies. Differences among waterfowl species in the number of diet studies in which seeds were carefully identified may have affected the chi-squared tests, where the differences in frequency distribution between the NWPL list and diet data were more likely to be statistically significant for well-sampled foraging groups (notably dabbling and diving ducks).

Ours is necessarily a qualitative study as we did not have quantitative data on the relative abundance of seeds from different plant species in waterfowl diet samples or in the wetland environment to properly test for selection on a local scale [47]. Differences in methods among diet studies made it impossible to extract comparable quantitative data [22]. In general terms, plants with smaller seeds release more seeds, and this could have influenced our results. For example, if there are more individual medium-sized seeds than large-sized seeds available in feeding patches, it might be that the apparent “preference” of waterfowl found for the former in our results is actually an effect of their availability. Even if this is the case, it does not change our finding that species with intermediate-sized seeds have a higher probability of ingestion and dispersal than species with larger seeds. Likewise, our results may have overstated the importance of terrestrial plant species, which were recorded at high frequencies in all waterfowl groups (although less than expected from the NWPL list), yet are often present in diet or fecal samples in lower abundance than aquatic or semiaquatic seeds [11,48]. Furthermore, we used seed ingestion as a proxy for seed dispersal and ignored the importance of plant traits in determining seed survival during gut passage as well as the gut retention time, which is also influenced by diet (see Green et al. [4,8] for a review). For example, plant species with smaller and/or harder seeds may show higher endozoochory rates due to greater seed survival during gut passage [31,49]. LDD events are favored by migratory behavior and by longer retention times (e.g., for larger seeds, [50]). Finally, our study compares waterfowl guilds, but there are also important differences among species within the same guild. By compiling additional trait data for plant species ingested by North American waterfowl, Almeida et al. [23] found evidence for important intraguild differences for dabbling ducks, sea ducks, and geese.

### 4.6. Future Research

More quantitative studies identifying seeds ingested and transported by waterfowl in North America would be beneficial (see also Green et al. [8], Almeida et al. [23]). More studies are needed that compare the availability of seeds in the environment with those that are ingested [1] or dispersed. Future experimental studies could quantify seed survival rates during waterfowl gut passage across traits (e.g., hardness and shape) and assess dispersal quality via germination trials [31,49]. Plant trait databases need improvement and lack information on traits known to be of importance for endozoochory, such as seed hardness and seed shape [49,50], as well as others that may also be potentially important to waterfowl (e.g., seed nutritional value, [29]).

## 5. Conclusions

Waterfowl ingest and disperse seeds of plant species from the NWPL across the range of trait values for moisture requirements, growth form, height, and seed mass. Their importance as LDD vectors has implications for how plants respond to climate change and land-use change as well as how non-indigenous plants can spread [8,11,16,50]. Endozoochory by waterfowl is not a random process at the continental scale across North American wetland ecosystems, and we have identified important patterns relating to waterfowl diet, wetland flora, and plant traits that could influence seed ingestion and dispersal. We found support for all four of our initial hypotheses with the selection of seeds from aquatic plants, but also of herbaceous graminoid growth forms. Plant height was not a limiting factor for seed ingestion and endozoochory, with the exception of diving ducks. Waterfowl showed selection for plants with seeds of an intermediate size.

Waterfowl guilds differ in seed selection, but the role that waterfowl species have as plant dispersers also depends on how the avian diet and other traits affect seed survival during gut passage [8] and how their migratory behavior [5,7], abundance [10], range, and distribution affect dispersal, all of which are changing in response to the profound climate and land-use changes underway in North America [51,52,53]. More studies of how traits can predict how seeds are selected for ingestion would facilitate wetland management for the benefit of waterfowl populations and would help us to predict how endozoochory by waterfowl may influence contemporary and future plant biogeography.

## Figures and Tables

**Table 1:** Summary of the chi-squared tests comparing the trait distributions of plant species on the NWPL list with those whose seeds were recorded as ingested by different waterfowl guilds (see also Figure 1). χ^2^ values are given for each guild. *** *p* < 0.001, ** *p* < 0.01, and * *p* < 0.05. Further details, including post hoc tests, are given in Tables S1–S8.

	Aquatic or Terrestrial	Growth Form	Plant Height Categories	Seed Mass Categories
Dabbling ducks	89.72 ***	24.45 ***	5.81	31.99 ***
Diving ducks	196.18 ***	31.60 ***	11.69 **	25.90 ***
Geese	25.55 ***	30.73 ***	6.65	11.19 *
Sea ducks	159.97 ***	7.38	4.41	12.53 **
Whistling ducks	0.21	39.46 ***	6.92	11.77 *

## Data Availability

All data sets in this paper are available in Almeida et al. [22], or in the Supplementary Materials.

## Supplementary Materials

The following supporting information can be downloaded at https://www.mdpi.com/article/10.3390/plants14131964/s1. File S1: Trait data from Díaz et al. [37], extracted for plant species whose seeds were identified from the digestive tracts or feces recovered from North American waterfowl (see Almeida et al. [22], for full details of plant seeds found in each waterfowl species). Plant species for which no trait data were available are not shown here. File S2: Trait data from Díaz et al. [37], extracted for plant taxa on the National Wetland Plant List [35]. File S3: Tables S1–S8. Table S1. Statistics, p value, observed and expected values, and residuals for the Chi-squared test comparing population probabilities of terrestrial, semiaquatic and aquatic plants in each foraging group and in the NWPL wetland plant list. Table S2. Post-hoc analyses based on residuals of Chi-squared Tests comparing population probabilities of terrestrial, semiaquatic and aquatic plants in each foraging group and in the NWPL wetland plant list. Table S3. Statistics, p value, observed and expected values, and residuals for the Chi-squared test comparing population probabilities of growth forms in each foraging group and in the NWPL wetland plant list. Table S4. Post-hoc analyses based on residuals of Chi-squared Tests comparing population probabilities of growth forms in each foraging group and in the NWPL wetland plant list. Table S5. Statistics, p value, observed and expected values, and residuals for the Chi-squared test comparing population probabilities of plant height categories in each foraging group and in the NWPL wetland plant list. Table S6. Post-hoc analyses based on residuals of Chi-squared Tests comparing population probabilities of plant height categories in each foraging group and in the NWPL wetland plant list. Table S7. Statistics, p value, observed and expected values, and residuals for the Chi-squared test comparing population probabilities of seed mass categories (mg) in each foraging group and in the NWPL wetland plant list. Table S8. Post-hoc analyses based on residuals of Chi-squared Tests comparing population probabilities of seed mass categories in each foraging group and in the NWPL wetland plant list.
