# Supplementary material for: Trait-Based Selection of Seeds Ingested and Dispersed by North American Waterfowl
Source: Plants (Basel). 2025 Jun 26;14(13):1964. doi: 10.3390/plants14131964 (PMC12251790; doi:10.3390/plants14131964)
Supplement: Supplementary file 1 [file plants-14-01964-s001.zip › plants-3680991-supplementary/File S3.pdf]

Table S1. Statistics, p value, observed and expected values, and residuals for the Chi-squared test comparing population probabilities of terrestrial, semiaquatic and aquatic plants in each foraging group and in the NWPL wetland plant list.

	χ^2	P value	Observed values		Expected values		Residuals	
			Foraging group	Wetlands	Foraging group	Wetlands	Foraging group	Wetlands
Dabbling ducks								
Aquatic	89.72	**0.00**	51.00	130.00	16.74	164.26	8.37	-2.67
Semiaquatic	89.72	**0.00**	8.00	27.00	3.24	31.76	2.65	-0.85
Terrestrial	89.72	**0.00**	317.00	3,533.00	356.03	3,493.97	-2.07	0.66
Diving ducks								
Aquatic	196.18	**0.00**	30.00	130.00	3.89	156.11	13.23	-2.09
Semiaquatic	196.18	**0.00**	3.00	27.00	0.73	29.27	2.66	-0.42
Terrestrial	196.18	**0.00**	59.00	3,533.00	87.38	3,504.62	-3.04	0.48
Geese								
Aquatic	25.55	**0.00**	6.00	130.00	1.42	134.58	3.84	-0.39
Semiaquatic	25.55	**0.00**	2.00	27.00	0.30	28.70	3.08	-0.32
Terrestrial	25.55	**0.00**	31.00	3,533.00	37.27	3,526.73	-1.03	0.11
Sea ducks								
Aquatic	159.97	**0.00**	17.00	130.00	1.65	145.35	11.93	-1.27
Semiaquatic	159.97	**0.00**	2.00	27.00	0.33	28.67	2.93	-0.31
Terrestrial	159.97	**0.00**	23.00	3,533.00	40.02	3,515.98	-2.69	0.29
Whistling ducks								
Aquatic	0.21	1.00	1.00	130.00	0.99	130.01	0.01	-0.00
Semiaquatic	0.21	1.00	0.00	27.00	0.20	26.80	-0.45	0.04
Terrestrial	0.21	1.00	27.00	3,533.00	26.81	3,533.19	0.04	-0.00

Table S2. Post-hoc analyses based on residuals of Chi-squared Tests comparing population probabilities of terrestrial, semiaquatic and aquatic plants in each foraging group and in the NWPL wetland plant list. P values below 0.05 are shown in bold.

statistic	Residuals		P values	
	Foraging group	Wetlands	Foraging group	Wetlands
Dabbling ducks				
Aquatic	**8.99**	**-8.99**	**0.00**	**0.00**
Semiaquatic	**2.79**	**-2.79**	**0.03**	**0.03**
Terrestrial	**-9.42**	**9.42**	**0.00**	**0.00**
Diving ducks				
Aquatic	**13.69**	**-13.69**	**0.00**	**0.00**
Semiaquatic	**2.70**	**-2.70**	**0.04**	**0.04**
Terrestrial	**-13.71**	**13.71**	**0.00**	**0.00**
Geese				
Aquatic	**3.93**	**-3.93**	**0.00**	**0.00**
Semiaquatic	**3.11**	**-3.11**	**0.01**	**0.01**
Terrestrial	**-4.91**	**4.91**	**0.00**	**0.00**
Sea ducks				
Aquatic	**12.24**	**-12.24**	**0.00**	**0.00**
Semiaquatic	**2.96**	**-2.96**	**0.02**	**0.02**
Terrestrial	**-12.46**	**12.46**	**0.00**	**0.00**
Whistling ducks				
Aquatic	0.01	-0.01	1.00	1.00
Semiaquatic	-0.45	0.45	1.00	1.00
Terrestrial	0.18	-0.18	1.00	1.00

Table S3. Statistics, p value, observed and expected values, and residuals for the Chi-squared test comparing population probabilities of growth forms in each foraging group and in the NWPL wetland plant list.

	χ^2	P value	Observed values		Expected values		Residuals	
			Foraging group	Wetlands	Foraging group	Wetlands	Foraging group	Wetlands
Dabbling ducks								
Other	24.45	**0.00**	16.00	156.00	16.87	155.13	-0.21	0.07
Herbaceous graminoid	24.45	**0.00**	127.00	784.00	89.35	821.65	3.98	-1.31
Herbaceous non-graminoid	24.45	**0.00**	183.00	1,846.00	199.01	1,829.99	-1.13	0.37
Shrub/tree	24.45	**0.00**	68.00	837.00	88.77	816.23	-2.20	0.73
Diving ducks								
Other	31.60	**0.00**	3.00	156.00	4.23	154.77	-0.60	0.10
Herbaceous graminoid	31.60	**0.00**	42.00	784.00	21.97	804.03	4.27	-0.71
Herbaceous non-graminoid	31.60	**0.00**	48.00	1,846.00	50.38	1,843.62	-0.34	0.06
Shrub/tree	31.60	**0.00**	6.00	837.00	22.42	820.58	-3.47	0.57
Geese								
Other	30.73	**0.00**	0.00	156.00	1.66	154.34	-1.29	0.13
Herbaceous graminoid	30.73	**0.00**	22.00	784.00	8.58	797.42	4.58	-0.48
Herbaceous non-graminoid	30.73	**0.00**	16.00	1,846.00	19.83	1,842.17	-0.86	0.09
Shrub/tree	30.73	**0.00**	1.00	837.00	8.92	829.08	-2.65	0.28
Sea ducks								
Other	7.38	0.06	3.00	156.00	1.99	157.01	0.71	-0.08
Herbaceous graminoid	7.38	0.06	11.00	784.00	9.97	785.03	0.33	-0.04
Herbaceous non-graminoid	7.38	0.06	29.00	1,846.00	23.51	1,851.49	1.13	-0.13
Shrub/tree	7.38	0.06	3.00	837.00	10.53	829.47	-2.32	0.26

Whistling ducks								
Other	39.46	**0.00**	0.00	156.00	1.24	154.76	-1.11	0.10
Herbaceous graminoid	39.46	**0.00**	20.00	784.00	6.38	797.62	5.39	-0.48
Herbaceous non-graminoid	39.46	**0.00**	9.00	1,846.00	14.73	1,840.27	-1.49	0.13
Shrub/tree	39.46	**0.00**	0.00	837.00	6.65	830.35	-2.58	0.23

Table S4. Post-hoc analyses based on residuals of Chi-squared Tests comparing population probabilities of growth forms in each foraging group and in the NWPL wetland plant list. P values below 0.05 are shown in bold.

statistic	Residuals		P values	
	Foraging group	Wetlands	Foraging group	Wetlands
Dabbling ducks				
Other	-0.23	0.23	1.00	1.00
Herbaceous graminoid	**4.77**	**-4.77**	**0.00**	**0.00**
Herbaceous non-graminoid	-1.70	1.70	0.71	0.71
Shrub/tree	-2.64	2.64	0.07	0.07
Diving ducks				
Other	-0.62	0.62	1.00	1.00
Herbaceous graminoid	**4.91**	**-4.91**	**0.00**	**0.00**
Herbaceous non-graminoid	-0.48	0.48	1.00	1.00
Shrub/tree	**-4.00**	**4.00**	**0.00**	**0.00**
Geese				
Other	-1.32	1.32	1.00	1.00
Herbaceous graminoid	**5.21**	**-5.21**	**0.00**	**0.00**
Herbaceous non-graminoid	-1.23	1.23	1.00	1.00
Shrub/tree	**-3.04**	**3.04**	**0.02**	**0.02**
Sea ducks				
Other	0.73	-0.73	1.00	1.00
Herbaceous graminoid	0.37	-0.37	1.00	1.00
Herbaceous non-graminoid	1.63	-1.63	0.82	0.82
Shrub/tree	-2.66	2.66	0.06	0.06
Whistling ducks				
Other	-1.14	1.14	1.00	1.00
Herbaceous graminoid	**6.13**	**-6.13**	**0.00**	**0.00**
Herbaceous non-graminoid	-2.14	2.14	0.26	0.26
Shrub/tree	**-2.95**	**2.95**	**0.03**	**0.03**

Table S5. Statistics, p value, observed and expected values, and residuals for the Chi-squared test comparing population probabilities of plant height categories in each foraging group and in the NWPL wetland plant list.

	χ^2	P value	Observed values		Expected values		Residuals	
			Foraging group	Wetlands	Foraging group	Wetlands	Foraging group	Wetlands
Dabbling ducks								
< 0.5	5.81	0.12	124.00	1,100.00	135.78	1,088.22	-1.01	0.36
0.5 - 1	5.81	0.12	98.00	640.00	81.87	656.13	1.78	-0.63
1 – 5	5.81	0.12	75.00	578.00	72.44	580.56	0.30	-0.11
> 5	5.81	0.12	48.00	447.00	54.91	440.09	-0.93	0.33
Diving ducks								
< 0.5	11.69	**0.01**	34.00	1,100.00	34.21	1,099.79	-0.04	0.01
0.5 - 1	11.69	**0.01**	25.00	640.00	20.06	644.94	1.10	-0.19
1 – 5	11.69	**0.01**	24.00	578.00	18.16	583.84	1.37	-0.24
> 5	11.69	**0.01**	3.00	447.00	13.57	436.43	-2.87	0.51
Geese								
< 0.5	6.65	0.08	17.00	1,100.00	13.57	1,103.43	0.93	-0.10
0.5 - 1	6.65	0.08	9.00	640.00	7.88	641.12	0.40	-0.04
1 – 5	6.65	0.08	8.00	578.00	7.12	578.88	0.33	-0.04
> 5	6.65	0.08	0.00	447.00	5.43	441.57	-2.33	0.26
Sea ducks								
< 0.5	4.41	0.22	20.00	1,100.00	17.15	1,102.85	0.69	-0.09
0.5 - 1	4.41	0.22	12.00	640.00	9.98	642.02	0.64	-0.08
1 – 5	4.41	0.22	9.00	578.00	8.99	578.01	0.00	-0.00
> 5	4.41	0.22	2.00	447.00	6.88	442.12	-1.86	0.23
Whistling ducks								
< 0.5	6.92	0.07	6.00	1,100.00	7.94	1,098.06	-0.69	0.06
0.5 - 1	6.92	0.07	8.00	640.00	4.65	643.35	1.55	-0.13
1 – 5	6.92	0.07	6.00	578.00	4.19	579.81	0.88	-0.08
> 5	6.92	0.07	0.00	447.00	3.21	443.79	-1.79	0.15

Table S6. Post-hoc analyses based on residuals of Chi-squared Tests comparing population probabilities of plant height categories in each foraging group and in the NWPL wetland plant list. P values below 0.05 are shown in bold.

statistic	Residuals		P values	
	Foraging group	Wetlands	Foraging group	Wetlands
Dabbling ducks				
< 0.5	-1.38	1.38	1.00	1.00
0.5 – 1	2.17	-2.17	0.24	0.24
1 – 5	0.36	-0.36	1.00	1.00
> 5	-1.08	1.08	1.00	1.00
Diving ducks				
< 0.5	-0.05	0.05	1.00	1.00
0.5 – 1	1.28	-1.28	1.00	1.00
1 – 5	1.57	-1.57	0.94	0.94
> 5	**-3.18**	**3.18**	**0.01**	**0.01**
Geese				
< 0.5	1.21	-1.21	1.00	1.00
0.5 – 1	0.46	-0.46	1.00	1.00
1 – 5	0.37	-0.37	1.00	1.00
> 5	-2.56	2.56	0.08	0.08
Sea ducks				
< 0.5	0.89	-0.89	1.00	1.00
0.5 – 1	0.73	-0.73	1.00	1.00
1 – 5	0.00	-0.00	1.00	1.00
> 5	-2.04	2.04	0.33	0.33
Whistling ducks				
< 0.5	-0.89	0.89	1.00	1.00
0.5 – 1	1.78	-1.78	0.60	0.60
1 – 5	1.00	-1.00	1.00	1.00
> 5	-1.96	1.96	0.40	0.40

Table S7. Statistics, p value, observed and expected values, and residuals for the Chi-squared test comparing population probabilities of seed mass categories (mg) in each foraging group and in the NWPL wetland plant list.

	χ^2	P value	Observed values		Expected values		Residuals	
			Foraging group	Wetlands	Foraging group	Wetlands	Foraging group	Wetlands
Dabbling ducks								
< 0.1	31.99	**0.00**	29.00	590.00	61.36	557.64	-4.13	1.37
0.1 – 1	31.99	**0.00**	112.00	1,106.00	120.74	1,097.26	-0.80	0.26
1 – 10	31.99	**0.00**	145.00	981.00	111.62	1,014.38	3.16	-1.05
10 – 100	31.99	**0.00**	49.00	377.00	42.23	383.77	1.04	-0.35
> 100	31.99	**0.00**	18.00	154.00	17.05	154.95	0.23	-0.08
Diving ducks								
< 0.1	25.90	**0.00**	9.00	590.00	14.93	584.07	-1.53	0.25
0.1 – 1	25.90	**0.00**	22.00	1,106.00	28.11	1,099.89	-1.15	0.18
1 – 10	25.90	**0.00**	46.00	981.00	25.60	1,001.40	4.03	-0.64
10 – 100	25.90	**0.00**	4.00	377.00	9.50	371.50	-1.78	0.29
> 100	25.90	**0.00**	1.00	154.00	3.86	151.14	-1.46	0.23
Geese								
< 0.1	11.19	**0.02**	0.00	590.00	6.37	583.63	-2.52	0.26
0.1 – 1	11.19	**0.02**	14.00	1,106.00	12.09	1,107.91	0.55	-0.06
1 – 10	11.19	**0.02**	16.00	981.00	10.76	986.24	1.60	-0.17
10 – 100	11.19	**0.02**	5.00	377.00	4.12	377.88	0.43	-0.05
> 100	11.19	**0.02**	0.00	154.00	1.66	152.34	-1.29	0.13
Sea ducks								
< 0.1	12.53	**0.01**	0.00	590.00	7.27	582.73	-2.70	0.30
0.1 – 1	12.53	**0.01**	12.00	1,106.00	13.77	1,104.23	-0.48	0.05
1 – 10	12.53	**0.01**	18.00	981.00	12.30	986.70	1.62	-0.18
10 – 100	12.53	**0.01**	8.00	377.00	4.74	380.26	1.50	-0.17
> 100	12.53	**0.01**	2.00	154.00	1.92	154.08	0.06	-0.01
Whistling ducks								
< 0.1	11.77	**0.02**	1.00	590.00	4.93	586.07	-1.77	0.16

0.1 – 1	11.77	**0.02**	13.00	1,106.00	9.34	1,109.66	1.20	-0.11
1 – 10	11.77	**0.02**	13.00	981.00	8.30	985.70	1.63	-0.15
10 – 100	11.77	**0.02**	0.00	377.00	3.15	373.85	-1.77	0.16
> 100	11.77	**0.02**	0.00	154.00	1.29	152.71	-1.13	0.10

Table S8. Post-hoc analyses based on residuals of Chi-squared Tests comparing population probabilities of seed mass categories in each foraging group and in the NWPL wetland plant list. P values below 0.05 are shown in bold.

statistic	Residuals		P values	
	Foraging group	Wetlands	Foraging group	Wetlands
Dabbling ducks				
< 0.1	**-4.79**	**4.79**	**0.00**	**0.00**
0.1 – 1	-1.03	1.03	1.00	1.00
1 – 10	**4.03**	**-4.03**	**0.00**	**0.00**
10 – 100	1.17	-1.17	1.00	1.00
> 100	0.25	-0.25	1.00	1.00
Diving ducks				
< 0.1	-1.72	1.72	0.86	0.86
0.1 – 1	-1.44	1.44	1.00	1.00
1 – 10	**4.92**	**-4.92**	**0.00**	**0.00**
10 – 100	-1.92	1.92	0.55	0.55
> 100	-1.51	1.51	1.00	1.00
Geese				
< 0.1	-2.81	2.81	0.05	0.05
0.1 – 1	0.68	-0.68	1.00	1.00
1 – 10	1.93	-1.93	0.54	0.54
10 – 100	0.46	-0.46	1.00	1.00
> 100	-1.33	1.33	1.00	1.00
Sea ducks				
< 0.1	**-3.00**	**3.00**	**0.03**	**0.03**
0.1 – 1	-0.59	0.59	1.00	1.00
1 – 10	1.96	-1.96	0.50	0.50
10 – 100	1.60	-1.60	1.00	1.00
> 100	0.06	-0.06	1.00	1.00
Whistling ducks				

< 0.1	-1.97	1.97	0.49	0.49
0.1 – 1	1.49	-1.49	1.00	1.00
1 – 10	1.97	-1.97	0.49	0.49
10 – 100	-1.90	1.90	0.58	0.58
> 100	-1.17	1.17	1.00	1.00
